# Neuromuscular blockers in the acute respiratory distress syndrome: A meta-analysis

**DOI:** 10.1371/journal.pone.0227664

**Published:** 2020-01-21

**Authors:** Yusi Hua, Xiaofeng Ou, Qian Li, Tao Zhu

**Affiliations:** 1 Department of Anesthesiology, West China Hospital of Sichuan University, Chengdu, Sichuan, China; 2 Department of Critical Care, West China Hospital of Sichuan University, Chengdu, Sichuan, China; East Carolina University Brody School of Medicine, UNITED STATES

## Abstract

**Background:**

The effects of neuromuscular blocking agents (NMBAs) on adult patients with acute respiratory distress syndrome (ARDS) remain unclear. We performed a meta-analysis of randomized controlled trials (RCTs) to evaluate its effect on mortality.

**Methods:**

We searched the Cochrane (Central) database, Medline, Embase, the Chinese Biomedical Literature Database (SinoMed), WanFang data and ClinicalTrials from inception to June 2019, with language restriction to English and Chinese. We included published RCTs and eligible clinical trials from ClinicalTrials.gov that compared NMBAs with placebo or usual treatment in adults with ARDS. We pooled data using random-effects models. The primary outcome was mortality. The secondary outcomes were the ratio of the partial pressure of arterial oxygen to the fraction of inspired oxygen (PaO_2_/FIO_2_), total positive end expiratory pressure (PEEP), plateau pressure (Pplat), days free of ventilator at day 28, barotrauma and ICU-acquired weakness.

**Results:**

We included 6 RCTs (n = 1557). Compared with placebo or usual treatment, NMBAs were associated with lower 21 to 28-day mortality (RR 0.72, 95% CI 0.53–0.97, I2 = 59%). NMBAs significantly improved oxygenation (Pao2:Fio2 ratios) at 48 hours (MD 27.26 mm Hg, 95% CI 1.67, 52.84, I2 = 92%) and reduced the incidence of barotrauma (RR 0.55, 95% CI 0.35, 0.85, I2 = 0). However, NMBAs had no effect on oxygenation (Pao2:Fio2 ratios) (MD 18.41 mm Hg, 95% CI -0.33, 37.14, I2 = 72%) at 24 hours. We also found NMBAs did not affect total PEEP, plateau pressure, days free of ventilation at day 28 and ICU-acquired weakness.

**Conclusions:**

In patients with moderate-to-severe ARDS, the administration of NMBAs could reduce 21 to 28-day mortality and barotrauma, and improve oxygenation at 48 hours, but have no significant effects on 90-day/ICU mortality, days free of ventilation at day 28 and the risk of ICU-acquired weakness. Further large-scale, high-quality RCTs are needed to confirm our findings. Registration: PROSPERO (ID: CRD 42019139656).

## Introduction

Acute respiratory distress syndrome (ARDS) is a life-threatening condition characterized by intense lung inflammation, consolidation, and progressive microatelectasis with refractory acute hypoxemia [1, 2]. Despite advances in medical equipments and clinical managements, the incidence and mortality of ARDS are still high [3–5]. Management of ARDS is a multimodal strategy involves non-pharmacologic interventions and pharmacologic interventions. Non-pharmacologic interventions include protective ventilation strategies, higher positive end-expiratory pressure (PEEP) and prone positioning, and these strategies are accepted because of their beneficial effects on patients with ARDS [2, 5, 6]. While, of the pharmacologic interventions, there are many inconclusive opinions remained.

The use of neuromuscular blocking agents (NMBAs) in ARDS has remained controversial. NMBAs were used in ICU mainly for facilitating lung-protective ventilation, preventing patient–ventilator dyssynchrony. Clinicians commonly consider NMBAs could reduce barotraumas, minimize the work of breathing and improve oxygenation [7–9]. In the early 2000s, a small, randomized trial conducted in France demonstrated continuous cisatracurium therapy could improve oxygenation of ARDS [8]. After two years, the same group of investigators conducted another randomized controlled trial (RCT) and founded cisatracurium could significantly reduce ARDS patients’ inflammatory biomarkers in both the blood and bronchoalveolar fluid, along with improved oxygenation [10]. In 2010, they reported a large multicenter trial (the ACURASYS trial) of 339 patients that the early administration of cisatracurium in patients with moderate-to-severe ARDS was associated with lower hospital mortality [11]. So, NMBAs have been recommended as clinical practice guideline for the management of severe ARDS and mechanical ventilation patients under certain circumstances [12, 13]. However, despite these encouraging results, the use of NMBAs did not be suggested as a clinical practice guideline for mechanical ventilation in adult patients with ARDS [14], and early neuromuscular blockade was also not widely adopted and strongly recommended in current guidelines [4, 15–17]. Resource constraints and limited data about the effects of NMBAs on neuromuscular function and other long-term outcomes may be the main potential concerns.

Some meta-analyses have reported that NMBAs play protective effects on ARDS patients [9, 18, 19]. However, the three included RCTs [8, 10, 11] were conducted in France by the same research group using the same NMBA, cisatracurium. In addition, some limitations including small sample sizes, poor quality trials and the narrative synthesis of data were prone to generate bias and heterogeneity. So, the results of the three meta-analyses should be reassessed. Moreover, recently, the largest multicenter, unblinded, randomized trial of 1006 patients with moderate-to-severe ARDS, the Reevaluation of Systemic Early Neuromuscular Blockade (ROSE) trial, was conducted by The Prevention and Early Treatment of Acute Lung Injury (PETAL) Clinical Trials Network of the National Heart, Lung, and Blood Institute (NHLBI). The investigators of that trial reported that there was no significant difference in mortality at 90 days between patients who received an early and continuous cisatracurium infusion and those who were treated with a usual-care approach with lighter sedation targets [20].

Therefore, based on these controversial findings related to NMBAs administration in adult patients with ARDS, we conducted a meta-analysis of RCTs to identify the benefits and adverse effects of NMBAs in ARDS patients.

## Methods

We conducted this study and reported the findings according to the guidelines recommended by the Cochrane Collaboration for Systematic Reviews of Interventions [21] and the Preferred Reporting Items for Systematic Reviews and Meta-Analyses (PRISMA) statement methodology [22] respectively. The protocol has been registered on PROSPERO (ID: CRD 42019139656).

### Literature search

We performed a computerized literature searches included Medline, Embase, CENTRAL (from inception to June 2019), the Chinese Biomedical Literature Database (SinoMed) (from 1978 to June 2019), and WanFang data (from 1990 to 2019), with language restriction to English and Chinese. We also searched ClinicalTrials.gov in June 2019 to identify additional eligible clinical trials for preliminary and unpublished results by contacting with authors if necessary. The exact search strategy is provided in S1 File.

### Study selection

After titles screening, we evaluated abstracts for relevance and identified them as included, excluded, or requiring further assessment. We considered randomized controlled studies (RCTs) eligible if they compared the administration of any NMBAs with placebo or usual treatment and included critical adult patients with ARDS, who were undergoing mechanical ventilation through an endotracheal tube and the ratio of the partial pressure of arterial oxygen (PaO_2_) to the fraction of inspired oxygen (FiO_2_) was less than 200 with the ventilator set to deliver a positive end-expiratory pressure of 5 cm of water or higher. We included studies only if a full text was available, with the interventions of interest were NMBAs, irrespective of the type, dose or duration. We excluded animal studies, observational studies, preclinical studies and trials of pediatric patients. We also excluded studies published in narrative reviews, commentaries, editorials and case reports.

### Data extraction and quality assessment

The primary outcome was mortality (included 21 to 28-day mortality and 90-day/ICU mortality). The secondary outcomes were the ratio of the partial pressure of arterial oxygen to the fraction of inspired oxygen (PaO_2_/FIO_2_) at 24 hours and 48 hours, total positive end expiratory pressure (PEEP) at 24 hours and 48 hours, plateau pressure (Pplat) at 24 hours and 48 hours, days free of ventilator at day 28, barotrauma (including pneumothorax, pneumomediastinum, pneumatocele, and subcutaneous emphysema) and ICU-acquired weakness.

Two investigators (Y.H. and X.O.) independently performed an initial screening according to titles or abstracts review, followed by a full-text screening. Detailed study information, study methods, methodologic quality, and outcomes were extracted using a standardized data extraction form. Disagreements were resolved by consensus or by a discussion with a third author (T.Z.). We assessed the risk of bias for each study using the Cochrane collaboration tool to judge the adequacy of random sequence generation, allocation concealment, blinding of participants and personnel, blinding of outcome assessment, incomplete outcome data, selective reporting and other bias [23]. The risk of bias in each of these domains was classified as high risk, low risk, or unclear. The overall risk of bias for an individual trial was categorized as low when the risk of bias was low in all domains; unclear when the risk of bias was unclear in at least one domain, with no high-risk domains; or high when the risk of bias was high in at least one domain [23].

### Statistical analysis

We performed all statistical analyses in the present study using Review Manager 5.3 (RevMan, The Cochrane Collaboration, Oxford, United Kingdom) and random-effects models. Risk ratios (RRs) with 95% confidence intervals (CIs) were calculated for binary outcomes, while mean differences (MDs) with 95% CIs were calculated for continuous outcomes. A *p* value of less than 0.05 was considered to be statistically significant. Data were obtained by direct extraction or by indirect calculation. We converted date reported as median and measure of dispersion to mean and standard deviation assuming a normal distribution by applying two simple formulae [24].

We assessed clinical and methodological heterogeneity according to the study characteristics and statistical heterogeneity by using the Mantel-Haenszel *χ*^*2*^ test and the *I*^2^ statistic. We considered heterogeneity to be substantial if the *I*^2^ value was 50% or greater or the *p* value was 0.1 or less [25, 26]. We assessed sensitivity analyses by using alternative effect measures (odds ratios v. risk ratios) and statistical models regarding heterogeneity (random v. fixed effects). For example, to assess the robustness of our primary results (mortality) in sensitivity analyses, we used fixed-effects models and two alternative statistical metrics: odds ratios and risk differences. Because of the different reported durations about mortality, PaO_2_/FIO_2_, total PEEP and Pplat, we analyzed mortality as tow subgroups, 21 to 28-day mortality and 90-day/ICU mortality, and we analyzed changes in PaO_2_/FIO_2_, total PEEP and Pplat at 24 and 48 hours. To assess the effect of NMBAs on the duration of ventilation, we analyzed days free of ventilator at day 28. We assessed the quality of evidence for clinical outcomes by using the Grading of Recommendations Assessment, Development and Evaluation (GRADE) approach [27]. Publication bias was not assessed because of the low power associated with the low number of included trails.

## Results

### Search results and study characteristics

**Fig 1:**
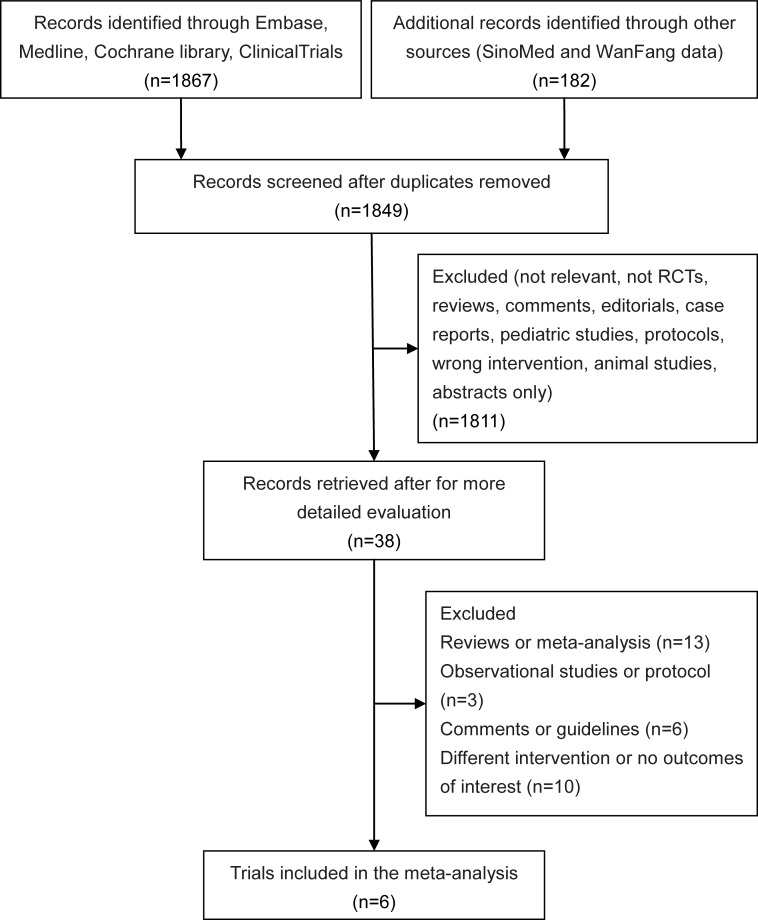
Search and selection of randomized controlled trials (RCTs).

We identified 2049 citations through electronic searches. After screening the titles and abstracts, we reviewed 38 studies in full, and 32 trials were excluded. The details of the 32 excluded trials and reasons for their exclusion are shown in S1 File. Finally, we included 6 RCTs [8, 10, 11, 20, 28, 29] with a total of 1557 patients in the meta-analysis (Fig 1).

The characteristics of the 6 included trials are shown in Table 1. Four of the trials included 455 patients (29.2%) were conducted in France by the same group of investigators [8, 11, 12, 29], the biggest trial was conducted in 48 hospitals across the United States with 1006 patients [20]. Population sizes of included studies varied greatly, ranging from 24 to 1006, and four trials included less than 100 patients [8, 10, 28, 29]. Most of the studies (5 trials included 1461 patients) were treated with cisatracurium [8, 10, 11, 20, 29], except one study performed in China received vecuronium [28]. All patients met the criteria for moderate to severe ARDS (the moderate to severe ARDS was defined as a baseline PaO_2_/FIO_2_≤ 200 mmHg [1]). The primary outcomes of three trials [11, 20, 28] were mortality, consistent with our study. The other three trials considered gas exchange (PaO_2_/FIO_2_ ratio) [1], inflammatory response [10] and transpulmonary pressures [29] as their primary outcomes respectively. Five trials reported 21 to 28-day mortality [8, 10, 20, 28], ICU/90-day mortality[8, 10, 20, 29], PaO_2_/FIO_2_ ratio at 24 hours[8, 10, 20, 29], PaO_2_/FIO_2_ ratio at 48 hours[8, 10, 20, 28, 29], total PEEP at 24 hours[8, 10, 11, 20, 29], Pplat at 24 hours[8, 10, 11, 20, 29] and days free of ventilation at day 28 [8, 10, 11, 20, 29]. Four trials reported total PEEP at 48 hours [8, 10, 20, 29], Pplat at 48 hours [8, 10, 20, 29], barotraumas [8, 10, 11, 20], and ICU-acquired weakness [8, 10, 11, 20].

**Table 1:** Characteristics of included trials.

Study	Setting	No. of patients (%, Male)	Age, yr, mean	Disease severity scores	Enrolment criteria	Experimental intervention	Control intervention
Gainner 2004 [8]	4 ICUs in France	56 (73.2)	NMBA: 59.8 Control: 61.5	SAPS II: NMBA:41.8 Control:45.4	ARDS PaO_2_:FiO_2_ ratio < 150 mm Hg and PEEP ≥ 5 cm H2O; Eligible < 36 hours;	A bolus of 50 mg cisatracurium followed by 5 μg∙kg−1∙min−1 infusion for 48 h.	An infusion of saline at a rate of 4 mL/h for control.
Forel 2006 [10]	3 ICUs in France	36(72.2)	NMBA: 52 Control: 61	SAPS II: NMBA:47 Control:49	ARDS PaO_2_:FiO_2_ ratio ≤ 200 mm Hg and PEEP ≥ 5 cm H2O; Eligible < 48 hours;	A bolus of 0.2 mg/kg cisatracurium followed by 5μg∙kg−1∙min−1 infusion for 48 h	An infusion of saline at a rate of 4 mL/h for control.
Papazian 2010 [11]	20 ICUs in France	339 (NA)	NMBA: 58 Control: 58	SAPS II: NMBA:50 Control:47	ARDS PaO_2_:FiO_2_ ratio < 150 mm Hg and PEEP ≥ 5 cm H2O; Eligible < 48 hours;	A bolus of 15 mg cisatracurium followed 37.5 mg∙h−1 for 48 h	A bolus of 15 mg placebo followed 37.5 mg∙h−1 for 48 h
Lyu 2014 [28]	1 ICUs in China	96 (66.7)	NMBA: 58.4 Control: 58.4	APACHE II: NMBA: 24.1 Control: 23.2	ARDS and sepsis PaO_2_:FiO_2_ ratio ≤ 200 mm Hg and PEEP ≥ 5 cm H2O; Eligible > 48 hours;	A bolus of 0.1 mg/kg vecuronium followed by 50 μg∙kg−1∙min−1 infusion for 24–48 h	Usual treatment
Guervilly 2017 [29]	2 ICUs in France	24 (79.2)	NMBA: 72 Control: 60	SAPS II: NMBA:47 Control:48	ARDS PaO_2_:FiO_2_ ratio < 150 mm Hg and PEEP ≥ 5 cm H2O; Eligible < 48 hours;	A bolus of 15 mg cisatracurium followed 37.5 mg∙h−1 for 48 h	Usual treatment
PETAL 2019 [20]	48 ICUs in the United States	1006 (55.7)	NMBA: 56.6 Control: 55.1	APACHE III: NMBA:103.9 Control:104.9	ARDS PaO_2_:FiO_2_ ratio < 150 mm Hg and PEEP ≥ 8g cm H2O; Eligible < 48 hours	A bolus of 15 mg cisatracurium followed 37.5 mg∙h−1 for 48 h	Usual treatment

ICU, intensive care unit; ARDS, Acute respiratory distress syndrome; NMBA, neuromuscular blocking agent; PEEP, positive end-expiratory pressure, APACHE, Acute Physiology and Chronic Health Evaluation; SAPS II, Simplified Acute Physiology Score II

**Fig 2:**
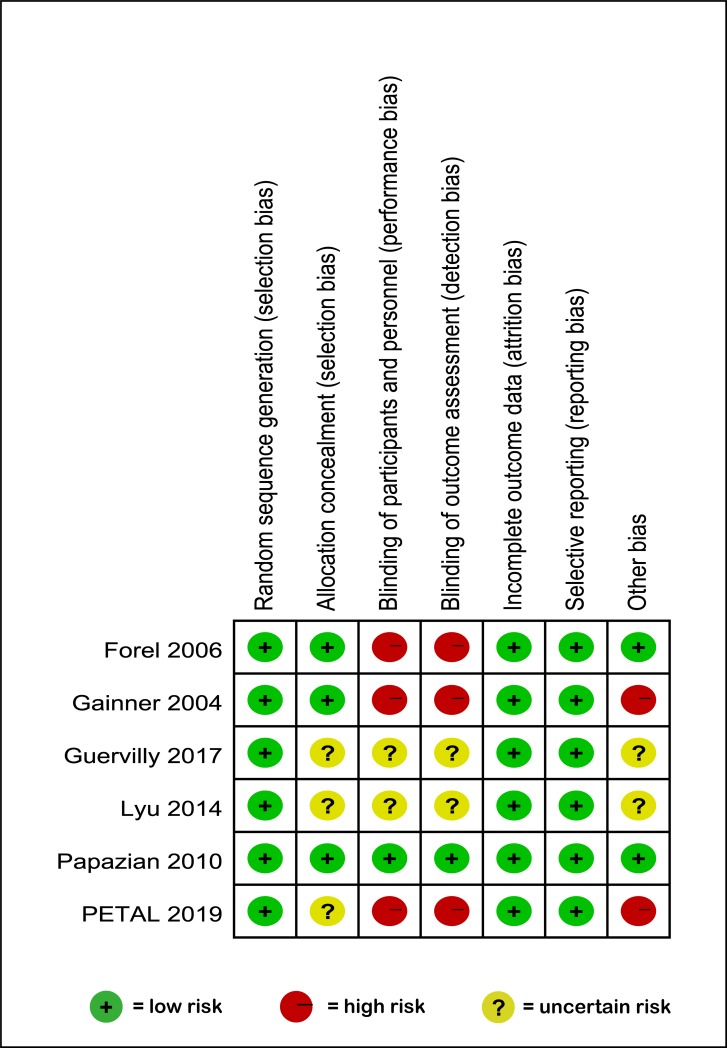
Risk of bias summary.

As shown in Table 2 and Fig 2, risks of bias were summarized by the Cochrane Risk of Bias tool; three trials [8, 10, 20] were judged to be at high risk of bias because of limitations in blinding; one trial was categorized as a lower risk of bias [11] with adequate randomized sequences, concealed allocation, blinding and completely reported clinical outcomes. Two trials [28, 29] were assessed to be unclear because insufficient data about whether blinding and concealed allocation were available. According to the GRADE approach, we judged the quality of evidence related to most of clinical outcomes included mortality, PaO2/FIO2 at 24 hours and at 48 hours, total PEEP at 24 hours and at 48 hours, Pplat at 24 hours and 48 hours, days free of ventilator at day 28 and barotrauma as moderate in light of the limitations in blinding and allocation concealment. We judged the quality of evidence related to ICU-acquired weakness as weak because of some imprecision.

**Table 2:** Risk of bias assessment.

Study	Sequence generation	Allocation concealment	Blinding	Incomplete outcome data	Selective reporting	Free of other bias	Overall risk of bias
Gainner 2004 [8]	Low: Computer-generated random number sequences	Low: Centralized	High: Nurses aware of assignment; infusion covered by sheet	Low: None	Low: None	Low: None	High
Forel 2006 [10]	Low: Computer-generated random number sequences	Low: Centralized	High: Nurses aware of assignment; infusion covered by sheet	Low: None	Low: None	Low: None	High
Papazian 2010 [11]	Low: Computer-generated random number sequences	Low: Centralized	Low: Blinding of patients, clinicians, evaluators, investigators, analysts	Low: None	Low: None	Low: None	Low
Lyu 2014 [28]	Low: with the random number table	Unclear	Unclear	Low: None	Low: None	Low: None	Unclear
Guervilly 2017 [29]	Low: Computer-generated random number sequences	Unclear	Unclear	Low: None	Low: None	Low: None	Unclear
PETAL 2019 [20]	Low: a permuted block design stratified by site	Unclear	High: unblinded	Low: None	Low: None	Low: None	High

Risk of bias was provided for each of the following domains: adequate random sequence generation; allocation sequence concealment; blinding for objective outcomes; incomplete outcome data; free of selective outcome reporting; free of other bias. Studies classed as at low risk of bias if all key domains were considered, high risk of bias if any one or more key domains were considered, Otherwise, they were considered as unclear risk of bias

### Mortality

#### Twenty-one to twenty-eight-day mortality

**Fig 3:**
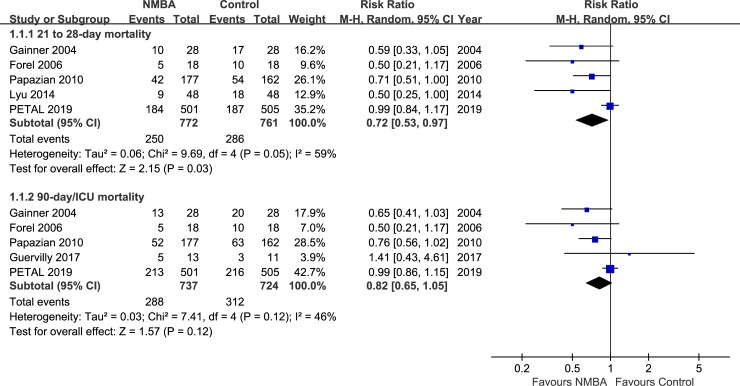
Comparison of mortality between the NMBAs group and Control group: 21 to 28-day mortality and 90-day/ICU mortality.

Five RCTs including 1,533 patients reported data on twenty-one to twenty-eight-day mortality with 32.38% in the NMBA group and 37.58% in the control group. Compared with the control oxygen group, there was a statistically significant reduction of mortality in the NMBA group (RR 0.72, 95% CI 0.53–0.97, *I*^2^ = 59%) (Fig 3). Considering four of the included trials were treated with cisatracurium, except the China trial [28] received vecuronium, we omitted the China trial and found that there was no significant difference between the NMBA group and the control group (RR 0.76, 95% CI 0.56–1.04, *I*^2^ = 58%) (S1 Fig).

#### Ninety-day/ICU mortality

In term of 90-day/ICU mortality, there were five trials included in this analysis with 1461 patients, which were treated with cisatracurium. We found no significant difference between the NMBA group and the control group (RR 0.82, 95% CI 0.65–1.05, *I*^2^ = 46%) (Fig 3).

#### Secondary outcomes

The secondary outcomes were summarized in Table 3. Considering the time effect of NMBAs, We analyzed PaO_2_/FIO_2_, total PEEP and Pplat at 24 hours and 48 hours respectively.

**Table 3:** Pooled analysis of secondary outcome measures.

Outcome measure	No. of trials (No. of patients)	Number of events in each group (%)	MD or RR (95% CI)	*I*^2^ value, %	*P* value
**PaO**_**2**_**:FiO**_**2**_ **ratio, mm Hg**					
At 24 hours	5 (1291)	n/a	18.41 (-0.33, 37.14)	72	0.05
At 48 hours	5 (941)	n/a	27.26 (1.67, 52.84)	92	0.04
**Total PEEP, cm of water**					
At 24 hours	5 (1407)	n/a	-0.20 (-0.86, 0.46)	55	0.55
At 48 hours	4 (1006)	n/a	-0.55 (-1.37, 0.28)	67	0.19
**Plateau pressureat, cm of water**					
At 24 hours	5 (1158)	n/a	0.05 (-0.97, 1.06)	42	0.93
At 48 hours	4 (761)	n/a	-0.08 (-0.90, 0.74)	0	0.85
**Days free of ventilation at day 28**					
	5 (1461)	n/a	0.72 (-0.49, 1.93)	12	0.24
**Barotrauma**					
	4 (1437)	NMBA: 29/724 (4.01%) Control: 52/713 (7.29%)	0.55 (0.35, 0.85)	0	0.008
**ICU-acquired weakness**					
	4 (1299)	NMBA: 50/659 (7.59%) Control: 39/640 (6.09%)	1.09 (0.76, 1.56)	0	0.63

ICU, intensive care unit; ARDS, Acute respiratory distress syndrome; NMBA, neuromuscular blocking agent; PEEP, positive end-expiratory pressure, PaO_2_:FiO_2_,the ratio of the partial pressure of arterial oxygen to the fraction of inspired oxygen

Data on PaO_2_/FIO_2_ ratios at 24 hours was available from 5 trials (n = 1291). No significant difference was found between the NMBA group and the control group (MD 18.41 mm Hg, 95% CI -0.33, 37.14, *I*^2^ = 72%). While at 48 hours, the pooled analysis from 5 trials with 941 patients suggested better PaO_2_/FIO_2_ in the NMBA group and the difference was statistically significant compared with the control group (MD 27.26 mm Hg, 95% CI 1.67, 52.84, *I*^2^ = 92%).

A total of 5 trials with 1407 patients and 4 trials with 1006 patients were included in the analysis of total PEEP at 24 hours and 48 hours respectively. No significant differences were found between the NMBA group and the control group neither at 24 hours (MD -0.20 cmH_2_O, 95% CI -0.86, 0.46, *I*^2^ = 55%) nor at 48 hours (MD -0.55 cmH_2_O, 95% CI -1.37, 0.28, *I*^2^ = 67%).

A total of 5 trials with 1158 patients and 4 trials with 761 patients were included in the analysis of Pplat at 24 hours and 48 hours respectively. No significant differences were found between the NMBA group and the control group neither at 24 hours (MD 0.05 cm H_2_O, 95% CI -0.97, 1.06, *I*^2^ = 42%) nor at 48 hours (MD -0.08 cm H_2_O, 95% CI -0.90, 0.74, *I*^2^ = 0).

With respect to the effects of NMBAs on days free of ventilator at day 28 (5 trials; n = 1461), we found no significant difference between the NMBA group and the control group (MD 0.72 days, 95% CI -0.49, 1.93, *I*^2^ = 12%).

About adverse events, 4 trials (n = 1437) described data on barotrauma. There were 29 (4.01%) patients who developed barotrauma in the NMBA group, 52 (7.29%) in the control group. Pooled analyses of the results showed it was significantly lower in the NMBA group than the control group (RR 0.55, 95% CI 0.35, 0.85, *I*^2^ = 0). Four trials (n = 1299) mentioned the incidence of ICU-acquired weakness. There were 50 (7.59%) patients in the NMBA group and 39 (6.09%) in the control group suffered ICU-acquired weakness, the difference was not significant (RR 1.09, 95% CI 0.76, 1.56, *I*^2^ = 0). There was no heterogeneity among the included trials about barotrauma and ICU-acquired weakness (*I*^2^ = 0, *p* = 0.70 and *I*^2^ = 0, *p* = 0.90, respectively).

### Sensitivity analysis

Sensitivity analyses by using alternative effect measures (odds ratios v. risk ratios) and statistical models regarding heterogeneity (random v. fixed effects) generated statistically similar primary results, with statistically significant reductions in 21 to 28-day mortality and similar effects about 90-day/ICU mortality between the NMBA group and the control group (S2–S4 Figs). The ROSE trial [20] was the largest and contributed the greatest weight to the results of our meta-analysis. By omitting the ROSE trial, we found that the effect of NMBA on 90-day/ICU mortality has changed from the same as the control group to statistically significant reduction effect. (RR, 0.72; 95% CI, 0.57, 0.91; *P* = 0.007; *I*^2^ = 0).

## Discussion

In this meta-analysis of randomized controlled trials for adult patients with moderate-to-severe ARDS, we found that the treatment of continuous infusion of NMBAs was associated with a lower risk of death at 21 to 28-day, but has no beneficial effects on 90-day/ICU mortality. Moreover, NMBAs treatment can improve PaO_2_/FIO_2_ at 48 hours, reduced the risk of barotrauma, and did not affect PaO2/FiO2 at 24 hours, total PEEP, plateau pressure, days free of ventilation at day 28 and ICU-acquired weakness.

Several systematic reviews and meta-analyses evaluated the effects of NMBAs on ARDS patients [9, 18, 19] and they all suggested that NMBAs treatment can improve outcomes in either primary or secondary measures. However, all the previous reviews and meta-analyses were mainly based on three RCTs [8, 10, 11], which were conducted in France by one research group and sued the same NMBA, cisatracurium. Different from previous studies, our meta-analysis had some characteristics. Firstly, this meta-analysis contains comprehensive outcomes: 21 to 28-day mortality, ICU/90-day mortality, PaO_2_/FIO_2_ ratio at 24 hours and 48 hours, total PEEP at 24 hours and 48 hours, Pplat at 24 hours and 48 hours, Days free of ventilation at day 28, barotraumas and ICU-acquired weakness. Secondly, based on the current available data, this study was the largest meta-analysis with 6 RCTs and 1557 patients. The sample sizes of previous studies were too small to accurate assessment of these outcomes, and the latest and largest RCT [20] could help to confirm the effects of NMBAs on ARDS. Inadequate number of patients and missing studies may affect the outcomes of NMBAs. Thirdly, in the present meta-analysis, we included the largest RCT [20] conducted in the United States and another RCT [28] using vecuronium, not cisatracurium, which may have substantial effects on the synthetic outcomes of NMBAs.

In terms of the 21 to 28-day mortality reduction associated with NMBAs therapy, there was high heterogeneity (chi^2^ = 9.69, df = 4, P = 0.05, *I*^2^ = 59%) among the included studies, which might because of the heterogeneous population and different NMBAs were used. One of the five included trials [28] conducted in China were treated with vecuronium. By omitting the China trial, we found that there was no significant difference between the NMBA group and the control group (RR 0.76, 95% CI 0.56–1.04, *I*^2^ = 58%). So, the decisive conclusion should be made cautiously and further large-scale, multicenter studies are needed to confirm the result. Our meta-analysis showed that there was no significant difference about 90-day/ICU mortality between patients treated with NMBA and patients not treated with NMBA. The use of NMBA usually requires deep sedation, and itself can result in negative outcomes [15, 30, 31]. Unlike other trials, the largest trial included in our study applied lighter sedation strategy in the control group, which may have decreased mortality in that group [20].

The improvements in the PaO_2_/FIO_2_ ratio at 48 hours and barotrauma were consistent with the results of some clinical studies and systematic researches [8, 9, 19, 32], although the potential mechanisms have not been entirely characterized. It is suggested that NMBAs block neuromuscular transmission of respiratory muscles, resulting in reducing patient-ventilator dyssynchrony, barotrauma, oxygen consumption, and the accumulation of alveolar fluid [7, 33, 34].

One of the main safety concerns with the administration of neuromuscular blocking agents is muscle weakness, sustained administration of neuromuscular blockade is associated with subsequent neuromuscular weakness [35, 36]. The risk of ICU-acquired weakness associated with NMBAs poses a strong resistance to NMBA usage in the current management of ARDS [32, 37]. Although NMBAs (cisatracurium besylate and vecuronium) have been reported with myopathy[32, 37], our study showed that there were 50 patients (7.59%) occurred ICU-acquired weakness in the NMBA group and 39 patients (6.1%) in the control group, and no significant difference was found between them (RR 1.09, 95% CI 0.76–1.56, *I*^2^ = 0%). This result maybe could be explained by the short duration of use of the neuromuscular blockades.

There are some limitations in our meta-analysis. First, our study was based on relatively few trials and 4 of them had small samples (< 100) [8, 10, 28, 29], which may have reduced precision and underestimated heterogeneity. In addition, different sedation strategy was applied in the control group, such as lighter sedation strategy conducted in the ROSE trial [20] may cause potential bias and heterogeneity. Second, different neuromuscular blockades may have different effects. An observational study compared cisatracurium and vecuronium reported that administration with cisatracurium was associated with more days free of ventilation and days not in ICU compared with vecuronium [38]. So, subgroup analyses stratified by different neuromuscular blockades even dosing strategies and specific groups of patients such as trauma, sepsis, pneumonia, and other causes should be planned and performed. Finally, most of the included trials were not double-blinded, because nurses, physiotherapists and other health care professionals were aware of the treatment assignments. The lack of double-blinding may have influenced short-term assessments of neuromuscular function, and the reporting of adverse events.

## Conclusion

In patients with moderate-to-severe ARDS, the administration of NMBAs could reduce 21 to 28-day mortality, but have no significant effect on 90-day/ICU mortality.

In addition, continuous infusion of NMBAs could improve PaO_2_/FIO_2_ ratios at 48 hours, reduce the incidence of barotrauma, without increasing days free of ventilation at day 28 and the risk of ICU-acquired weakness. The effects of NMBAs on ARDS patients should be re-evaluated.

## Supporting information

S1 Checklist PRISMA checklist. (DOC)

S1 Fig Comparison of mortality in four included trials treated with cisatracurium. (EPS)

S2 Fig Sensitivity analysis of mortality by using alternative effect measure risk ratio and fixed effects model in randomized controlled trials. (EPS)

S3 Fig Sensitivity analysis of intubation rate by using alternative effect measure odds ratio and random effects model in randomized controlled trials. (EPS)

S4 Fig Sensitivity analysis of intubation rate by using alternative effect measure odds ratio and fixed effects model in randomized controlled trials. (EPS)

S1 File Details of search strategy and excluded studies. (DOCX)
