# Supplementary material for: Neuromuscular blockers in the acute respiratory distress syndrome: A meta-analysis
Source: PLoS One. 2020 Jan 21;15(1):e0227664. doi: 10.1371/journal.pone.0227664 (PMC6974254; doi:10.1371/journal.pone.0227664)
Supplement: S1 File — (DOCX) [file pone.0227664.s006.docx]

**Details of Search Strategy and Excluded Studies**

**Search Strategy**

**Ovid MEDLINE(R) (From 1946 to 04 June 2019)**

1. Acute Respiratory Distress Syndrome.mp. or Respiratory Distress Syndrome, Adult/

2. Respiratory Distress Syndrome, Adult/ or ARDS.mp. or Acute Lung Injury/

3. exp lung diseases/

4. Respiratory Distress*.mp. or Respiratory Insufficiency/

5. idiopathic respiratory distress syndrome.mp.

6. transfusion related acute lung injury.mp.

7. shock lung.mp.

8. human ards.mp.

9. noncardiogenic pulmonary edema.mp.

10. increased-permeability pulmonary edema.mp.

11. stiff lung.mp.

12. acute respiratory distress.mp.

13. (acute lung injury or ALI).mp.

14. pneumonia.mp.

15. hyaline membrane disease.mp.

16. exp respiratory insufficiency/

17. (respiratory adj1 (failure or insufficiency)).mp.

18. Or/1-17

19. exp Neuromuscular Blocking Agents/

20. Neuromuscular Blocking Agent*.mp.

21. Neuromuscular Blocker*.mp.

22. Neuromuscular Blocking drug*.mp.

23. (Rapacuronium or Raplon or Mivacurium or Mivacron or Atracurium or Tracrium or Doxacurium or Nuromax or Cisatracurium or Nimbex or Vecuronium or Norcuron or Rocuronium or Zemuron or Pancuronium or Pavulon or Tubocurarine or Jexin or gallamine or Flaxedil or Pipecuronium or alcuronium orcurare or toxiferine).mp.

24. Or/19-23

25. 18 and 24

**EMBASE (From 1974 to 06 June 2019)**

1. Acute Respiratory Distress Syndrome.mp. or Respiratory Distress Syndrome, Adult/

2. Respiratory Distress Syndrome, Adult/ or ARDS.mp. or Acute Lung Injury/

3. exp lung diseases/

4. Respiratory Distress*.mp. or Respiratory Insufficiency/

5. idiopathic respiratory distress syndrome.mp.

6. transfusion related acute lung injury.mp.

7. shock lung.mp.

8. human ards.mp.

9. noncardiogenic pulmonary edema.mp.

10. increased-permeability pulmonary edema.mp.

11. stiff lung.mp.

12. acute respiratory distress.mp.

13. (acute lung injury or ALI).mp.

14. pneumonia.mp.

15. hyaline membrane disease.mp.

16. exp respiratory insufficiency/

17. (respiratory adj1 (failure or insufficiency)).mp.

18. 1 or 2 or 3 or 4 or 5 or 6 or 7 or 8 or 9 or 10 or 11 or 12 or 13 or 14 or 15 or 16 or 17

19. exp Neuromuscular Blocking Agents/

20. Neuromuscular Blocking Agent*.mp.

21. Neuromuscular Blocker*.mp.

22. Neuromuscular Blocking drug*.mp.

23. (Rapacuronium or Raplon or Mivacurium or Mivacron or Atracurium or Tracrium or Doxacurium or Nuromax or Cisatracurium or Nimbex or Vecuronium or Norcuron or Rocuronium or Zemuron or Pancuronium or Pavulon or Tubocurarine or Jexin or gallamine or Flaxedil or Pipecuronium or alcuronium orcurare or toxiferine).mp.

24. 19 or 20 or 21 or 22 or 23

25. 18 and 24

26. limit 25 to randomized controlled trial

**CENTRAL (May 2019)**

1. Acute Respiratory Distress Syndrome.mp. or Respiratory Distress Syndrome, Adult/

2. Respiratory Distress Syndrome, Adult/ or ARDS.mp. or Acute Lung Injury/

3. exp lung diseases/

4. Respiratory Distress*.mp. or Respiratory Insufficiency/

5. idiopathic respiratory distress syndrome.mp.

6. transfusion related acute lung injury.mp.

7. shock lung.mp.

8. human ards.mp.

9. noncardiogenic pulmonary edema.mp.

10. increased-permeability pulmonary edema.mp.

11. stiff lung.mp.

12. acute respiratory distress.mp.

13. (acute lung injury or ALI).mp.

14. pneumonia.mp.

15. hyaline membrane disease.mp.

16. exp respiratory insufficiency/

17. (respiratory adj1 (failure or insufficiency)).mp.

18. 1 or 2 or 3 or 4 or 5 or 6 or 7 or 8 or 9 or 10 or 11 or 12 or 13 or 14 or 15 or 16 or 17

19. exp Neuromuscular Blocking Agents/

20. Neuromuscular Blocking Agent*.mp.

21. Neuromuscular Blocker*.mp.

22. Neuromuscular Blocking drug*.mp.

23. (Rapacuronium or Raplon or Mivacurium or Mivacron or Atracurium or Tracrium or Doxacurium or Nuromax or Cisatracurium or Nimbex or Vecuronium or Norcuron or Rocuronium or Zemuron or Pancuronium or Pavulon or Tubocurarine or Jexin or gallamine or Flaxedil or Pipecuronium or alcuronium orcurare or toxiferine).mp.

24. 19 or 20 or 21 or 22 or 23

25. 18 and 24

**ClinicalTrials.gov (06 June 2019)**

Neuromuscular Blocking Agents | acute Respiratory Distress Syndrome | Start date on or before 06/07/2019

Also searched for Respiratory Distress Syndrome, Acute Respiratory Distress, Acute lung injury and more

**Chinese Biomedical Literature Database (SinoMed) (From 1978 to 2019)**

("急性呼吸窘迫综合征"[全字段:智能]) OR "急性呼吸衰竭"[全字段:智能]) AND （"神经肌肉阻滞剂"[全字段:智能]) OR "肌松药"[全字段:智能] OR "肌松剂"[全字段:智能]）

**WanFang data (From 1990 to 2019)**

全部:(全部:(神经肌肉阻滞剂)+全部:(肌松药)+全部:(肌松剂))*全部:(全部:(急性呼吸窘迫综合征)+全部:(急性呼吸衰竭))

**Reference list of excluded articles**

**Systematic review or meta-analysis (13)**

1 Duggal A, Ganapathy A, Ratnapalan M, Adhikari NK. Pharmacological treatments for acute respiratory distress syndrome: systematic review. Minerva Anestesiol. 2015; 81:567-88.

2 Yegneswaran B, Murugan R. Neuromuscular blockers and ARDS: thou shalt not breathe, move, or die! Crit Care. 2011; 15:311.

3 Torbic H, Duggal A. Neuromuscular blocking agents for acute respiratory distress syndrome. [Review]. Journal of Critical Care. 2019; 49:179-84.

4 Wang AT, Gao JL, Li XL, Leng YX, Yao ZY, Zhu X. [The effect of neuromuscular blocking agents on prognosis of patients with acute respiratory distress syndrome: a meta analysis]. [Chinese]. Zhonghua Wei Zhong Bing Ji Jiu Yi Xue. 2013; 25:149-53.

5 Tao W, Yang LQ, Gao J, Shao J. Neuromuscular blocking agents for adult patients with acute respiratory distress syndrome: A meta-analysis of randomized controlled trials. The Journal of Trauma and Acute Care Surgery. 2018; 85:1102-9.

6 Price DR, Mikkelsen ME, Umscheid CA, Armstrong EJ. Neuromuscular Blocking Agents and Neuromuscular Dysfunction Acquired in Critical Illness: A Systematic Review and Meta-Analysis. Crit Care Med. 2016; 44:2070-8.

7 Neto AS, Pereira VG, Esposito DC, Damasceno MC, Schultz MJ. Neuromuscular blocking agents in patients with acute respiratory distress syndrome: a summary of the current evidence from three randomized controlled trials. Annals of Intensive Care. 2012; 2:26.

8 Syed A, Kobzik A, Huang DT. Role of Pharmacologic Paralysis in Acute Respiratory Distress Syndrome. Semin Respir Crit Care Med. 2019; 40:101-13.

9 Tsai-Nguyen G, Modrykamien AM. Use of neuromuscular blocking agents in acute respiratory distress syndrome. [Review]. Baylor University Medical Center Proceedings. 2018; 31:177-9.

10 Paramore S. Effects of the use of neuromuscular blocking agents on acute respiratory distress syndrome outcomes: A systematic review. [Review]. Journal of the American Association of Nurse Practitioners. 2018; 30:327-32.

11 Hraiech S, Forel JM, Papazian L. The role of neuromuscular blockers in ARDS: benefits and risks. [Review]. Current Opinion in Critical Care. 2012; 18:495-502.

12 Bourenne J, Hraiech S, Roch A, Gainnier M, Papazian L, Forel JM. Sedation and neuromuscular blocking agents in acute respiratory distress syndrome. Ann Transl Med. 2017; 5:291.

13 Tonelli AR, Zein J, Adams J, Ioannidis JP. Effects of interventions on survival in acute respiratory distress syndrome: an umbrella review of 159 published randomized trials and 29 meta-analyses. [Review]. Intensive Care Medicine. 2014; 40:769-87.

**Observational studies or protocol (3)**

14 Sottile PD, Kiser TH, Burnham EL, Ho PM, Allen RR, Vandivier RW, et al. An Observational Study of the Efficacy of Cisatracurium Compared with Vecuronium in Patients with or at Risk for Acute Respiratory Distress Syndrome. American Journal of Respiratory & Critical Care Medicine. 2018; 197:897-904.

15 Feng, B, Mao, ZR. Efect of early application of neuromuscular blocking agents on the treatment of severe ARDS patients with mechanical ventilation. Chinese Journal of Clinical Research. 2016; 29 :305-307.

16 Huang DT, Angus DC, Moss M, Thompson BT, Ferguson ND, Ginde A, et al. Design and Rationale of the Reevaluation of Systemic Early Neuromuscular Blockade Trial for Acute Respiratory Distress Syndrome. Annals of the American Thoracic Society. 2017; 14:124-33.

**comments or guidelines (6)**

17 Needham CJ, Brindley PG. Best evidence in critical care medicine: The role of neuromuscular blocking drugs in early severe acute respiratory distress syndrome. Canadian Journal of Anaesthesia. 2012; 59:105-8.

18 Huang DT, Papazian L. Is Cisatracurium the Neuromuscular Blocking Agent of Choice in Acute Respiratory Distress Syndrome? Am J Respir Crit Care Med. 2018; 197:849-50.

19 Coggeshall JW, Marini JJ, Newman JH. Improved oxygenation after muscle relaxation in adult respiratory distress syndrome. Archives of Internal Medicine. 1985; 145:1718-20.

20 Bolaki M, Amargianitakis V, Georgopoulos D, Guervilly C, Papazian L: Effects of neuromuscular blockers on transpulmonary pressures in moderate to severe acute respiratory distress syndrome. In. *Intensive Care Medicine 43(4):600-601, 2017 04.* 2017.

21 Sevransky J. ACP Journal Club. 48 hours of cisatracurium reduced 90-day mortality in patients with early, severe ARDS. Ann Intern Med. 2011; 154:JC1-3.

22 Murray MJ, DeBlock H, Erstad B, Gray A, Jacobi J, Jordan C, et al. Clinical Practice Guidelines for Sustained Neuromuscular Blockade in the Adult Critically Ill Patient. Crit Care Med. 2016; 44:2079-103.

**Different intervention or no outcomes of interest (10)**

23 Light RW, Bengfort JL, George RB. The adult respiratory distress syndrome and pancuronium bromide. Anesth Analg. 1975; 54:219-23.

24 Hraiech S, Forel JM, Guervilly C, Rambaud R, Lehingue S, Adda M, et al. How to reduce cisatracurium consumption in ARDS patients: the TOF-ARDS study. Annals of Intensive Care. 2017; 7.

25 Matte Al, Ferguson ND, Slutsky AS, Arroliga AC, Cook DJ, Douglas IS, et al. Patterns of neuromuscular blockade in an international trial of HFO for adults with ARDs. American journal of respiratory and critical care medicine. 2012; 185.

26 Barmparas G, Dhillon NK, Tatum JM, Patel K, Thomsen GM, Mason R, et al. Extended neuromuscular blockade in acute respiratory distress syndrome does not increase mortality. J Surg Res. 2018; 231:434-40.

27 Lefrant JY, Farenc C, De la Coussaye JE, Muller L, Ripart J, Cuvillon P, et al. Pharmacodynamics and atracurium and laudanosine concentrations during a fixed continuous infusion of atracurium in mechanically ventilated patients with acute respiratory distress syndrome. Anaesth Intensive Care. 2002; 30:422-7.

28 Moore L, Kramer CJ, Delcoix-Lopes S, Modrykamien AM. Comparison of Cisatracurium Versus Atracurium in Early ARDS. Respiratory Care. 2017; 62:947-52.

29 Macario A, Chow JL, Dexter F. A Markov computer simulation model of the economics of neuromuscular blockade in patients with acute respiratory distress syndrome. BMC Medical Informatics & Decision Making. 2006; 6:15.

30 Slack DF, Corwin DS, Shah NG, Shanholtz CB, Verceles AC, Netzer G, et al: Pilot Feasibility Study of Therapeutic Hypothermia for Moderate to Severe Acute Respiratory Distress Syndrome. In. *Crit Care Med 2017 Jul;45(7):1152-1159.* 2017.

31 Torbic H, Bauer SR, Personett HA, Dzierba AL, Stollings JL, Ryder LP, et al. Perceived safety and efficacy of neuromuscular blockers for acute respiratory distress syndrome among medical intensive care unit practitioners: A multicenter survey. Journal of Critical Care. 2017; 38:278-83.

32 Dhonneur G, Cerf C, Lagneau F, Mantz J, Gillotin C, Duvaldestin P. The pharmacokinetics of cisatracurium in patients with acute respiratory distress syndrome. Anesthesia & Analgesia. 2001; 93:400-4.
